# The Regulation of the Z- and G-Box Containing Promoters by Light Signaling Components, SPA1 and MYC2, in *Arabidopsis*

**DOI:** 10.1371/journal.pone.0062194

**Published:** 2013-04-30

**Authors:** Sreeramaiah N. Gangappa, Jay P. Maurya, Vandana Yadav, Sudip Chattopadhyay

**Affiliations:** 1 National Institute of Technology, Durgapur, India; 2 National Institute of Plant Genome Research, New Delhi, India; University of Nottingham, United Kingdom

## Abstract

Although many transcription factors and regulatory proteins have been identified and functionally characterized in light signaling pathways, photoperception to transcription remains largely fragmented. The Z-box is one of the LREs (Light responsive elements) that plays important role in the regulation of transcription during light-controlled Arabidopsis seedling development. The involvement of photoreceptors in the modulation of the activity of the Z-box containing promoters has been demonstrated. However, the role of downstream signaling components such as SPA1 and MYC2/ZBF1, which are functionally interrelated, remains unknown. In this study, we have investigated the regulation of the Z-box containing synthetic and native promoters by SPA1 and MYC2 by using stable transgenic lines. Our studies suggest that SPA1 negatively regulates the expression of *CAB1* native promoter. MYC2 negatively regulates the activity of Z- and/or G-box containing synthetic as well as native promoters irrespective of light quality. Moreover, MYC2 negatively regulates the expression of *Z/G-NOS101-GUS* even in the darkness. Furthermore, analyses of tissue specific expression in adult plants suggest that MYC2 strongly regulates the activity of Z- and G-box containing promoters specifically in leaves and stems. In roots, whereas MYC2 positively regulates the activity of the Z-box containing synthetic promoter, it does not seem to control the activity of the G-box containing promoters. Taken together, these results provide insights into SPA1- and MYC2-mediated transcriptional regulation of the Z- and G-box containing promoters in light signaling pathways.

## Introduction

Light plays a pivotal role in the growth and development of plants starting from seed germination to de-etiolation of seedlings, pigment synthesis, chloroplast differentiation, flowering and senescence [1]–[3]. Light modulates the gene expression primarily at the transcriptional level [4]. Many of the light-controlled developments are triggered by changes in the gene expression through the regulation of transcription of specific genes in defined tissue types and at various developmental stages [5]–[10]. Photomorphogenesis is one of the well-studied photo-responses in *Arabidopsis*. In dark, seedlings undergo skotomorphogenesis (etiolation), which is characterized by long hypocotyl, apical hook and development of proplastids into etioplasts, by contrast light grown seedlings show a characteristic pattern of development called photomorphogenesis (de-etiolation), with short hypocotyl, open cotyledons, well developed chloroplasts and de-repression of light inducible genes [2]–[4]. During the switch from skotomorphogenesis to photomorphogenesis, transcriptional reprogramming of a large number of genes occurs in Arabidopsis. Many of the photosynthetic machinery related genes are expressed during the shift from dark to light [11], [12]. Genetic, biochemical and mutational studies of Arabidopsis seedling development have identified several genes, which function downstream to phytochrome, cryptochrome or both the signaling pathways [2], [4], [13]. SPA1 functions as a negative regulator in far-red light, and can suppress *phyA* phenotype [13], whereas MYC2 is a bHLH transcription factor that works as a negative regulator in cryptochrome-mediated blue light signaling [14].

*SPA1* belongs to a gene family that includes the other members such as *SPA2, SPA3* and *SPA4* [15]–[17]. Biochemically, SPA1 helps COP1, a ubiquitin ligase, in the ubiquitylation of target proteins including HY5, HFR1 and LAF1 [18]–[22]. Recent studies show that MYC2 binds to the G-box LRE (light responsive element) of *SPA1* promoter and regulates its expression in a COP1 dependent manner [23]. SPA1 has been shown to negatively regulate the expression of light inducible genes such as *CAB1*, *CAB3* and *CHS* in dark and light adapted seedlings [22], [24], [25]. Further, SPA1 has been reported to regulate flowering under short day photoperiod by negatively regulating the expression of *FT* transcript levels indirectly by degrading CO protein [26].

Analyses of the promoter sequences of light-inducible genes have led to the identification of multiple cis-acting regulatory elements, also known as LREs [27]–[30]. There are at least four commonly occurring LREs: G, GATA, GT1 and Z-box, which have been demonstrated to be essential for the regulation of light-mediated transcriptional activity [5], [6], [28]–[30], [31]–[35]. Recent studies have identified and functionally characterized several Z-box binding factors (ZBFs) including ZBF1/MYC2, ZBF2/GBF1 and ZBF3/CAM7 [14], [23], [36]–[39]. The ZBFs have been shown to interact with both the Z- and G-box LREs present in the light regulated promoters [14], [36], [37]. All these studies indicate that the Z- and G-box are functionally equivalent with context to MYC2 mediated gene regulation. In this study, we have investigated the functional relevance of interaction of MYC2 with the Z- and G-box containing promoters. We have also investigated the regulation of the Z-box containing promoters by SPA1 during early seedling development. Our results suggest that whereas SPA1 strongly represses Z-box containing native *CAB1* promoter, it strongly promotes the activity of Z-box containing synthetic promoter in the roots. Further, MYC2 negatively regulates the activity of Z- and/or G-box containing synthetic as well as native promoters in dark and different light qualities in the seedling stage. However, in adult plants MYC2 differentially regulates the expression of these promoters in a tissue specific and promoter context manner. Collectively, our results provide an insight for the regulation of Z-box LRE containing promoters and their transcriptional regulation mediated by MYC2 and SPA1.

## Materials and Methods

### Plant Materials and Growth Conditions

All the promoter-reporter constructs used in this study have been described in Puente *et al*. [33], except *4G/NOS101-GUS*, which was generated by genetic crosses between Col-0 and *hy5-215* containing *4G/NOS101-GUS*. Selected stable transgenes were individually introduced into s*pa1-2* [15] and *atmyc2-3/zbf1-1* [14] mutants by genetic crosses with the wild-type transgenic lines. The mutant lines homozygous for each transgene were obtained from the F3 generation for further studies. Putative transgenic plants were screened histochemically for verification of the expression of *uidA* gene [40]. Seeds were surface-sterilized and plated on MS medium supplemented with 0.8% Bactoagar (Difco). The plates were then cold-treated at 4°C for 3 days and then transferred to light chambers maintained at 22°C with the desired wavelength and intensity of light. For all monochromatic light assays, the plates were transferred to continuous white light for 3 h to induce germination. The plates were then transferred to monochromatic light conditions, incubated at 22°C for six days. For the growth of *Arabidopsis* seedlings, the white light intensity used was 90 µmol m^−2^ sec^−1^. For the color light sources the intensities used (in LED chamber: Q-beam 3200-A; Quantum Devices, inc., WI 53507, USA) were, far-red light of 60 µmol m^−2^ sec^−1^, red light of 90 µmol m^−2^ sec^−1^ and blue light of 30 µmol m^−2^ sec^−1^.

### GUS Histochemical Staining and Assay

GUS staining (using about 40–50 seedlings in each sample) were carried out following the same procedure as mentioned [29]. Wild-type and mutant plants (about 20–30 seedlings each) containing the same transgene were stained for the same length of time. Putative transgenic plants were screened histochemically for verification of the expression of *uidA* gene. Histochemical assay for GUS was carried out in the intact tissues (organ or whole seedlings or free hand cut sections). GUS histochemical assay/GUS spectrometric assay were carried out using six-day-old seedlings or 35-day-old adult transgenic plants grown under required conditions. Tissue from the control and transgenic plants were submerged in fixation buffer (2% formaldehyde, 50 mM sodium phosphate (pH 7.0), 0.05% Triton X-100), and vacuum infiltrated for 4 to 5 min on ice and kept at room temperature for 10 min. The fixation buffer was removed and the material was washed twice with 50 mM sodium phosphate buffer (pH 7.0) to remove fixative buffer. The tissue samples were stained using staining buffer (1.5 mM of X-gluc, 50 mM sodium phosphate (pH 7.0) and 0.1% Triton X-100) by vacuum infiltrating for 5 to 10 min and then wrapped with aluminium foil and incubated at 37°C overnight in darkness. After staining, tissue was bleached extensively with 70% ethanol to remove the chlorophyll. Representative pictures were photographed.

### GUS Spectrometric Assay

For GUS spectrometric assays six-day-old seedlings grown in dark and different wavelengths of light or 35-day-old adult plant-parts were harvested in microcentrifuge tube and snap freeze in liquid nitrogen and ground in 1 ml of extraction buffer [50 mM sodium phosphate (pH 7.0), 5 mM DTT, 1 mM EDTA, 0.1% sarcosyl, 0.1% Triton X- 100] at 4°C. The suspension was transferred into a fresh tube and 50 µl of supernatant was added to the 450 µl of assay buffer (1 mM MUG in extraction buffer) and incubated in 37°C for 30 min. GUS activity was determined by fluorimetric assay as described by Jefferson (1987) in which MUG was used as a substrate. Total protein was quantified using the Bradford solution and GUS specific activity was recorded as nanomoles of 4-MU formed per milligram of protein per hr from the initial velocity of the reaction [40]. Finally the GUS activity was calculated by comparing the reading to the MU standard and normalizing to the total protein content. The experiment was done at least in three biological and three technical replicates.

## Results

### The Activation of the Z-box Containing Promoters is Altered in *spa1* Mutants

**Figure 1:**
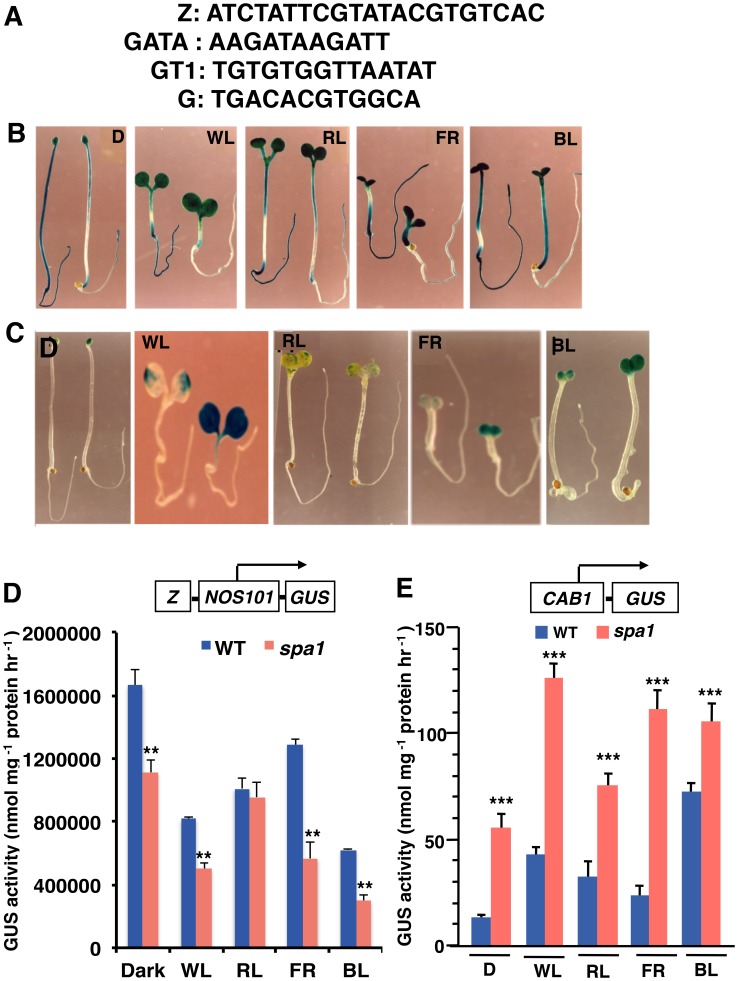
Effect of *spa1* mutation on the regulation of *Z/NOS101*–*GUS* and *CAB1-GUS* promoters under different wavelengths of light. (**A**)**,** The consensus DNA sequences of LREs (Z, GATA, GT1 and G-box) derived from different light responsive promoters. (**B–C**)**,** In each panel, wild-type (WT) and *spa1* mutant seedlings carrying respective transgene were shown on the left and right, respectively. GUS staining patterns of 6-day-old wild-type and *spa1* mutant seedlings carrying *Z/NOS101-GUS* (**B**) and *CAB1-GUS* (**C**) transgene grown in different light (white light (WL), far-red light (FR), red light (RL), and blue light (BL) or dark (D)) conditions as indicated. (**D**)**,** GUS activities of six-day-old constant D, WL, RL, FR and BL grown seedlings carrying *Z/NOS101-GUS* transgene in wild-type and *spa1* mutant backgrounds. Error bars represents SD (n = 3). ** P≤0.01 for values significantly differ from corresponding light conditions in wild-type. (**E**)**,** GUS activities of six-day-old constant D, WL, RL, FR and BL grown seedlings carrying *CAB1-GUS* transgene in wild-type and *spa1* mutant backgrounds. Error bars represents SD (n = 3). *** P≤0.001 for values significantly differ from corresponding light conditions in wild-type. All the above experiments were performed at least thrice with similar results.

SPA1 has been reported to negatively regulate the expression of *CAB3* and *CHS* transcript levels in dark grown seedlings in phyA dependent manner [24], and *CAB3*, *CHS* and *RBCS* expression in far-red light (FR) adapted seedlings [22], [24]. Also, SPA1 negatively regulates the accumulation of *CAB1* in dark and blue light (BL) adapted seedlings [25]. While many light inducible promoters are active in *spa1* mutants, the role of SPA1 in the regulation of the Z-box containing promoters remains unknown. We therefore asked whether the activity of the Z-box containing promoters is affected in the regulatory pathways defined by *spa1* mutation. We used stable transgenic lines containing *Z/NOS101-GUS* and *CAB1-GUS* transgene for this study (Figure 1A). The basal promoter used in the synthetic promoters has been taken from the nopaline synthase gene (*NOS101*), which is from −101 to +4, contains the CAAT and the TATA boxes, and is not active in transgenic plants [33], [41], [42]. Earlier studies have shown that paired-element, but not the single-element, containing synthetic promoters can mimic the native light regulated promoters [36]. All these promoter-reporter constructs (Figure 1A) were individually introduced into *spa1* mutant background by genetic crosses with the wild type transgenic lines. Then the mutant lines homozygous for each transgene were generated for further studies.

We monitored the activity of the Z-box containing promoters as reflected by the GUS reporter enzymatic activity measurements. The expression of *Z-NOS101* promoter was detected in all the organs including cotyledons, hypocotyl and roots of wild-type seedlings either grown in dark or at various wavelengths of light (Figure 1B). In *spa1* mutants, however, the expression of the transgene was mostly confined to cotyledon and hypocotyl with very little expression, if any, in root (Figure 1B). Quantification of the GUS activity measurements revealed that the activity of the promoter was significantly reduced in *spa1* mutants as compared to wild-type in dark and at various wavelengths of light, except red light (RD) (Figure 1D). Collectively, these results indicate that SPA1 is required for the optimum activation of the *Z-NOS101* synthetic promoter.

To further test these observations, we used native *CAB1* minimal promoter (*CAB1-GUS*) that contains a single Z-box LRE that is critical for its activation [28], [31], [33], [42]. The GUS activity staining of *CAB1* promoter in wild-type and *spa1* mutant backgrounds revealed that the *CAB1* promoter activity was confined to cotyledons at various wavelengths of light (Figure 1C). Whereas no activity of *CAB1* promoter was detceted in wild-type background, *CAB1-GUS* expression was clearly visible in *spa1* mutants in dark. The quantification of GUS activity revealed that the promoter activity was stronger in *spa1* mutants than the wild-type seedlings in dark, white light (WL), RL, FR and BL (Figure 1E).Quantification of GUS activity measurements revealed that the *CAB1* promoter activity was ∼2 to 4-fold increased in *spa1* in dark, WL, RL and BL, whereas ∼5-fold increased in FR as compared to wild-type (Figure 1E). These results indicate that SPA1 negatively regulates the activity of *CAB1* promoter in dark and at various wavelengths of light.

### MYC2/ZBF1 Negatively Regulates the Activity of the Z-box Containing Promoters

**Figure 2:**
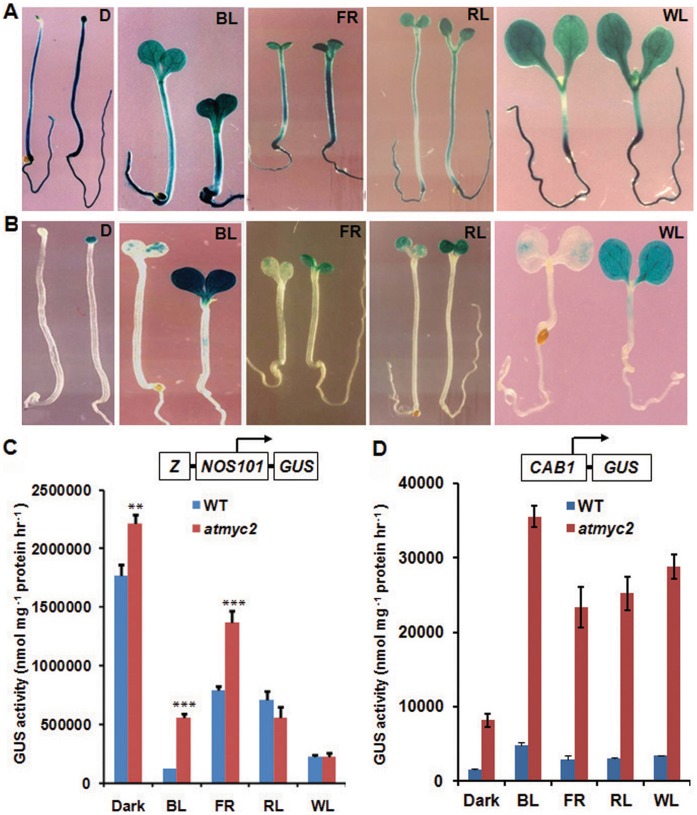
Effect of *atmyc2/zbf1* mutation on the regulation of Z-box containing promoters. (**A–B**)**,** In each panel, wild-type and *atmyc2/zbf1* mutant seedlings carrying respective transgene were shown on the left and right, respectively. GUS staining patterns of six-day-old wild-type and *atmyc2* seedlings carrying *Z/NOS101-GUS* (**A**) and *CAB1-GUS* (**B**) transgene grown in different light or dark conditions as indicated. (**C–D**)**,** GUS activities of wild-type and *atmyc2* seedlings carrying *Z/NOS101-GUS* (**C**) and *CAB1-GUS* (**D**) transgene grown in different light or dark conditions as indicated. Error bars represents SD (n = 3). ** P≤0.01 and *** P≤0.001 for values significantly differ from WT in respective growth conditions. All the above experiments were performed at least thrice with similar results.

DNA-protein interaction studies have earlier shown that MYC2 interacts with the Z-box of *CAB1* minimal promoter [14]. The expression of *CAB1* is also dramatically elevated in *atmyc2* mutant seedlings in BL and FR [14]. However, it remains unknown whether the activity of the Z-box containing promoter is directly affected in the regulatory pathways defined by MYC2 *in planta*. To determine how MYC2 is involved in the regulation of Z-box containing promoters, we used stable transgenic lines containing *Z/NOS101-GUS* and *CAB1-GUS* promoter-reporter constructs [42]. These promoter-reporter constructs were individually introduced into *atmyc2-3* mutants by genetic crosses with the wild type transgenic lines. Mutant lines homozygous for each transgene were then generated for further studies. We used 6-day-old seedlings grown in constant dark or at different wavelengths of light to monitor the activity of *Z/NOS101* and *CAB1* promoters. Similar to wild type, *Z/NOS101-GUS* transgene was expressed in various tissues in *atmyc2* mutants in D, BL, FR, RL and WL (Figures 2A). The quantitative GUS activity measurements revealed that the activity of *Z/NOS101* promoter was significantly increased in *atmyc2* background as compared to wild type seedlings in D, BL and FR (Figure 2C). However, no noticeable difference in the activity between WT and *atmyc2* mutant was observed in RL and WL (Figure 2C). To further test this observation, we used native *CAB1-GUS* promoter-reporter construct. Earlier studies revealed that in wild type background the expression of *CAB1-GUS* was confined to the cotyledons in light, and was not detectable in any tissue-type of dark grown seedlings [35], [42]. In *atmyc2* mutant background, the expression of *CAB1-GUS* transgene was although mostly confined to the cotyledons, the level of expression was dramatically elevated compared to wild-type seedlings in BL, FR and RL and WL (Figure 2B). Most strikingly, *CAB1-GUS* transgene was expressed in the cotyledons of *atmyc2* mutant seedlings even in the darkness (Figure 2B). Quantitative GUS activity measurements revealed that ∼4-fold higher activity of *CAB1* promoter was present in *atmyc2* mutants than wild-type background in dark (Figure 2D). Similarly, ∼5 to 7 fold increased activity of *CAB1* promoter was detected in *atmyc2* mutants compared to wild-type seedlings in BL, FR, RL or WL (Figure 2D). Taken together, these results suggest that MYC2 represses the activity of the Z-box containing promoters at different wavelengths of light.

### MYC2 Strongly Represses Light Mediated Induction of *CAB1* Promoter Activity

**Figure 3:**
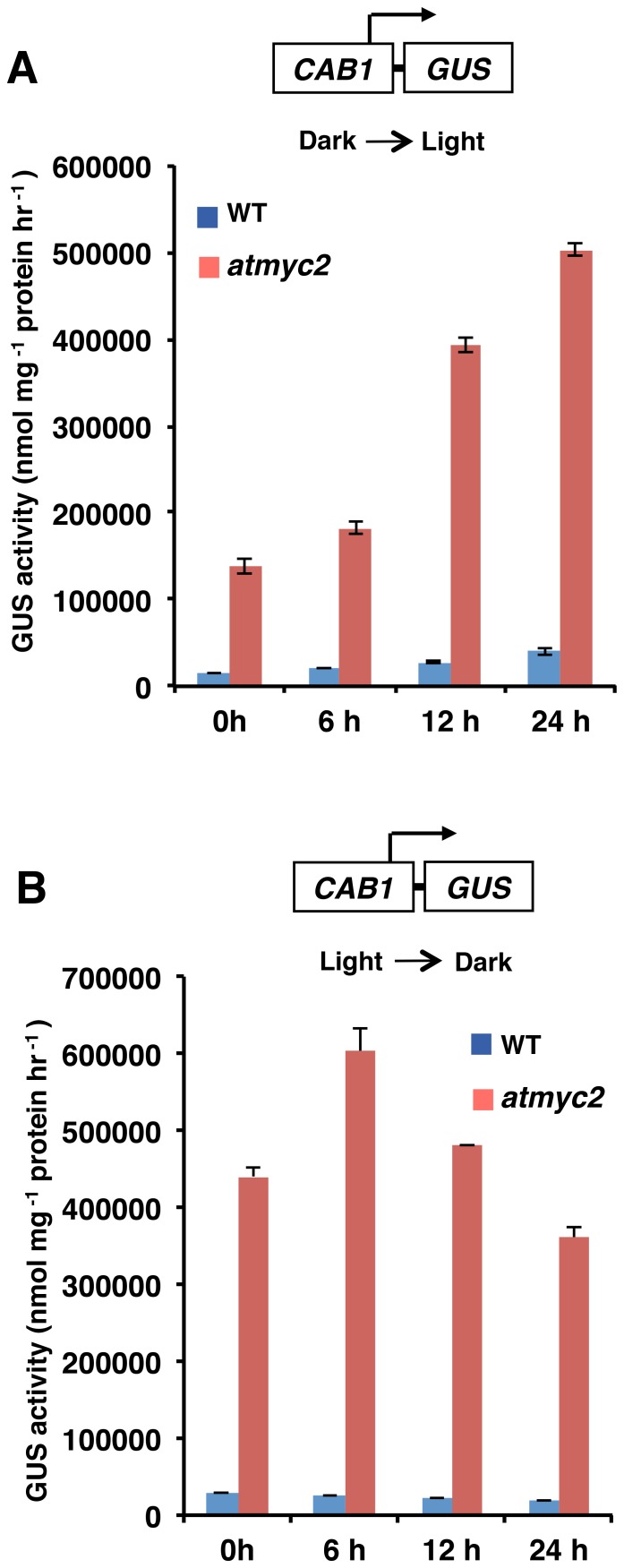
Light-mediated induction of *CAB1-GUS* in *atmyc2* mutatants. (**A**)**,** Four-day-old dark-grown seedlings carrying *CAB1-GUS* transgene were exposed to WL for 0, 6, 12 and 24 h and GUS activities were measured. Error bars represents SD (n = 3). (**B**)**,** Four-day-old WL grown seedlings carrying *CAB1-GUS* transgene were exposed to dark for 0, 6, 12 and 24 h and GUS activities were measured**.** Error bars represents SD (n = 3). All the above experiments were performed at least thrice with similar results.

As *CAB1* promoter was found to be more active in dark and light grown seedlings in *atmyc2* mutant background, we wanted to examine the light or dark mediated induction of *CAB1* promoter during the transition from light to dark and *vice-versa* in *atmyc2* mutants. We monitored the induction kinetics of *CAB1* promoter in light and dark-adapted seedlings in wild-type and *atmyc2* mutant backgrounds. As shown in Figure 3, when 4-day-old dark grown seedlings were exposed to WL for 0, 6, 12 and 24 h of WL, the *CAB1* promoter activity was found to be gradually induced with the increase in exposure to WL. On the other hand, the rate of induction of *CAB1* promoter was drastically increased in *atmyc2* mutants (Figure 3A). Next, to examine the activity of the *CAB1* promoter during light to dark transition, we transferred 4-day-old WL grown seedlings to dark for 0, 6, 12 and 24 h. As shown in Figure 3B, at 6 h although *CAB1* promoter activity was reduced in wild-type background, the activity of the promoter was further increased in *atmyc2* mutant. Whereas at 12 h of dark exposure the *CAB1* promoter activity was found to be similar to constant WL grown *atmyc2* mutant seedlings, at 24 h the *CAB1* promoter activity was significantly reduced in comparison to 12 h (Figure 3B). Collectively, these results demonstrate that MYC2 is a strong repressor of *CAB1* promoter activity.

### MYC2/ZBF1 Negatively Regulates the Activity of the G-box Containing Promoters

**Figure 4:**
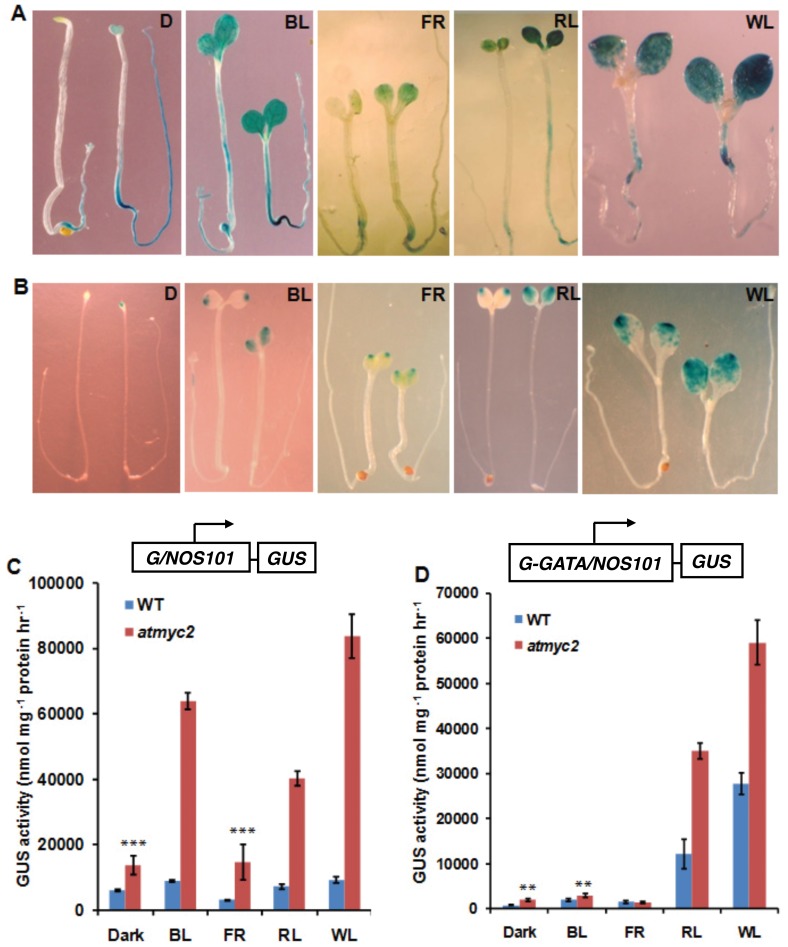
Effect of *atmyc2/zbf1* mutation on the regulation of G-box containing promoters. (**A–B**)**,** In each panel, wild-type and *atmyc2/zbf1* mutant seedlings carrying respective transgene were shown on the left and right, respectively. GUS staining patterns of six-day-old wild-type and *atmyc2* seedlings carrying *G/NOS101-GUS* (**A**) and *G-GATA/NOS101-GUS* (**B**) transgene grown in different light or dark conditions as indicated. (**C–D**) GUS activities of wild-type and *atmyc2* seedlings carrying *G/NOS101-GUS* (**C**) and *G-GATA/NOS101-GUS* (**D**) transgene grown in different light or dark conditions as indicated. Error bars represents SD (n = 3). ** P≤0.01 and *** P≤0.001 for values significantly differ from WT in respective growth conditions. All the above experiments were performed at least thrice with similar results.

The G-box and the Z-box have been shown to be functionally equivalent with context to MYC2-mediated regulation. DNA-protein interaction studies of MYC2 with G-box have shown that MYC2 also interacts with the G-box of *RBCS-1A* minimal promoter [14]. However, it is not yet known whether the activity of the G-box containing promoters is directly affected by mutations in *MYC2*. To investigate the regulatory role of MYC2 on G-box containing promoters, we used stable transgenic lines containing *G/NOS101-GUS* and *G-GATA/NOS101-GUS* [28], [33] promoter-reporter constructs. Both these promoter-reporter constructs were individually introduced into *atmyc2-3* mutants by genetic crosses with the wild-type transgenic lines. Mutant lines homozygous for each transgene were then generated; and 6-day-old seedlings grown in constant dark D, BL, FR, RL and WL were used for this study. The expression of *G/NOS101-GUS* transgene was mostly detected in cotyledons with lesser extents in hypocotyl and root of both wild-type and *atmyc2* mutants. However, the level of expression of the transgene was increased in the *atmyc2* mutants in dark and all light conditions tested (Figure 4A). The quantitative GUS activity measurements revealed that the activity of *G/NOS101* promoter was increased to about ∼6 to 8-fold in BL and WL, and ∼2 to 4-fold in D, FR and RL in *atmyc2* as compared to wild-type seedlings (Figure 4C). Collectively, these results suggest that MYC2 represses the activity of the *G/NOS101* promoter in dark and various wavelengths of light.

Expression of *G-GATA/NOS101-GUS* transgene was confined to cotyledons and the intensity of the GUS stain was increased in the *atmyc2* mutants in dark, BL, RL and WL (Figure 4B). The quantitative GUS activity measurements revealed that the activity of *G-GATA/NOS101* promoter was significantly increased in dark, BL, RL and WL with ∼2 to 4-fold higher in RL and WL compared to wild-type (Figure 4D).

### MYC2/ZBF1 Differentially Regulates Z- and G-box Containing Promoters in Tissue Specific Manner

**Figure 5:**
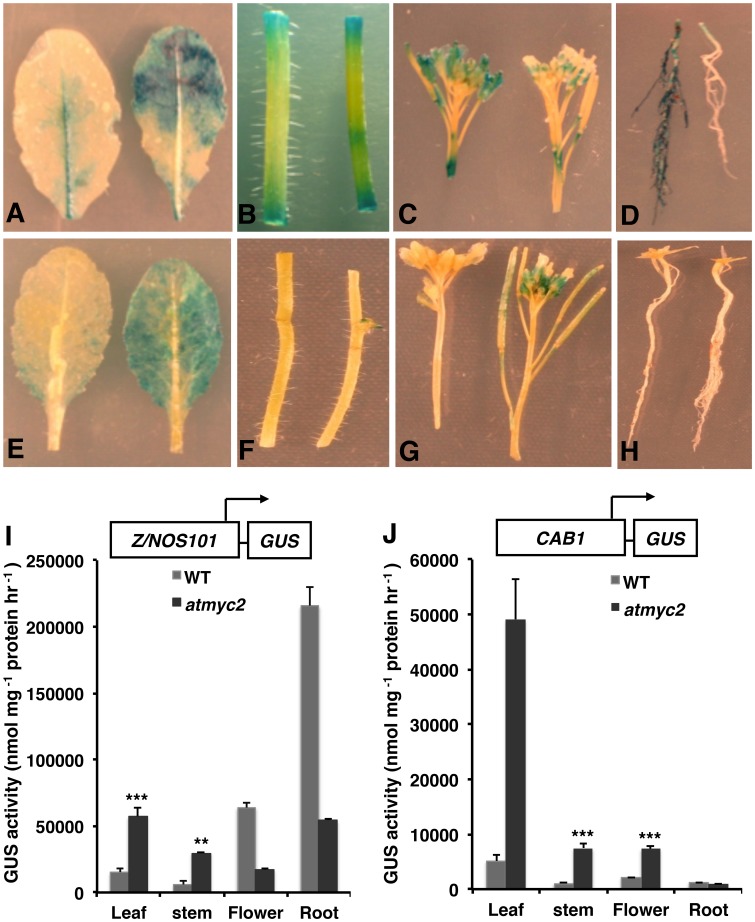
Effect of *atmyc2/zbf1* mutation on the tissue specific expression of Z- box containing promoters in adult plants. In each panel (A-H) wild-type and *atmyc2* seedlings carrying respective transgene are shown on the left and right, respectively. For tissue specific staining, 35-days-old adult plants grown in 14 h Light/10 h Dark cycle were used for the experiment. (**A–D**) The GUS staining patterns of *Z/NOS-GUS* transgene from leaf (A), stem (B), flower (C) and root (D). (**E–H**) The GUS staining patterns of *CAB1-GUS* transgene from leaf (E), stem (F), flower (G), and root (H). (**I–J**) GUS activities of 35-day-old wild-type and *atmyc2* plants carrying *Z/NOS101-GUS* (**I**) and *CAB1-GUS* (**J**) transgene. Error bars represents SD (n = 3). Error bars represents SD (n = 3). ** P≤0.01 and *** P≤0.001 for values significantly differ from WT in respective tissues. All the above experiments were performed at least thrice with similar results.

The *MYC2* mutant plants display delayed flowering with less number of lateral roots; and the adult plants have short stature as compared to corresponding wild-type [14]. To examine the tissue specific regulatory role of *MYC2* in adult plants, we grew wild-type and *atmyc2* mutant transgenic plants in 16 h light/8 h dark cycles. When the plants started forming inflorescence (35-day-old), different parts of the plant (stem, leaf, flower and root) were stained and simultaneously measured the GUS activities. The GUS staining results revealed that, the *Z/NOS101* promoter activity was increased in leaves and stems (Figure 5A–B), whereas it was decreased in flower and roots in *atmyc2* mutants compared to wild-type plants (Figure 5C–D). The quantitative GUS activity results show that *Z/NOS101* promoter activity was maximum in roots as compared to other organs of the plants. However, the activity of *Z/NOS101* promoter was strongly suppressed in *atmyc2* mutants in roots (Figure 5I). GUS activity measurements also revealed significant enhancement of the promoter activity in leaf and stem in *atmyc2* plants as compared to wild-type (Figure 5I). Taken together, these results suggest that, MYC2 plays both negative (leaf and stem) and positive (flower and root) regulatory roles for *Z/NOS101* promoter activity in the adult plants. We then extended our study to native *CAB1* minimal promoter. The *CAB1-GUS* transgene was expressed in leaves and sepals as revealed by GUS activity staining in wild type and *atmyc2* mutants (Figure 5E and G). Whereas no *CAB1* promoter activity was detected in stem or root of wild-type plants, the branching points of the stems displayed the activity of the promoter in *atmyc2* mutants (Figure 5F and H). However, no activity was detected in roots of the *atmyc2* mutant plants similar to wild type (Figure 5H). The activity of *CAB1* promoter was found to be stronger in *atmyc2* mutants as compared to wild-type plants in leaf, stem and flower (Figure 5J). These results suggest that MYC2 negatively regulates the activity of *CAB1* promoter in adult plants.

**Figure 6:**
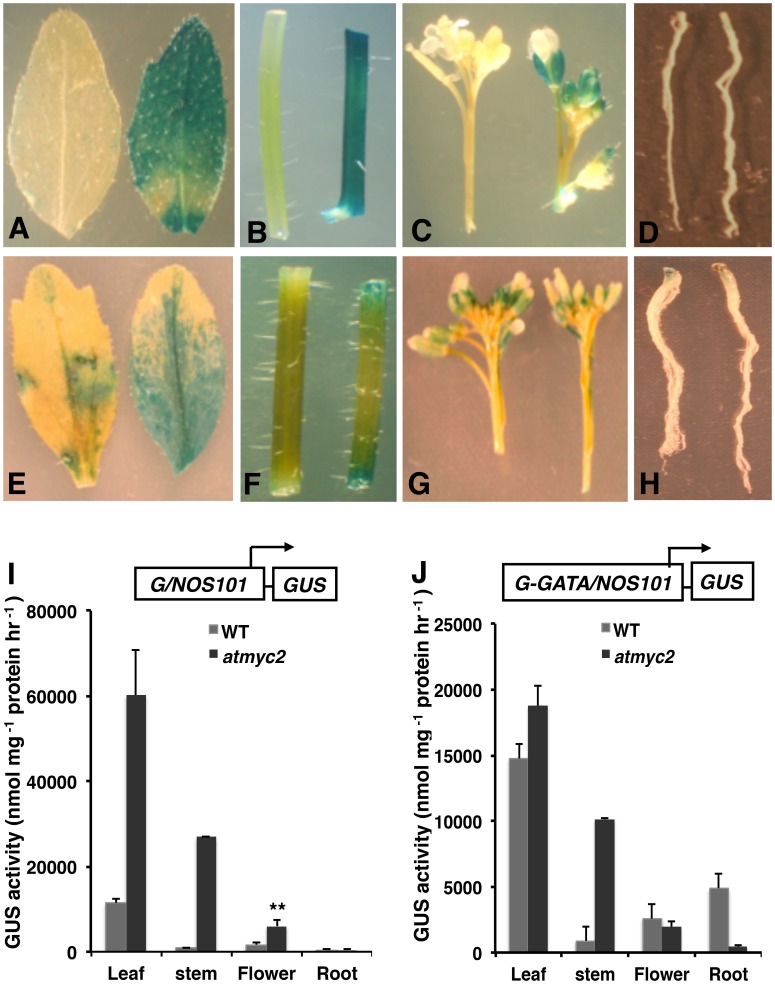
Effect of *atmyc2/zbf1* mutation on the tissue specific expression of G- box containing promoters in adult plants. (**A–D**)The GUS staining patterns of *G/NOS101-GUS* transgene from leaves (A), stem (B),flower (C), and root (D). (**E–H**)The GUS staining patterns of *G-GATA/NOS101-GUS* transgene from leaves (E), stem (F), flower (G), and root (H). (**I–J**) Comparison of GUS activities of 35-day-old wild-type and *atmyc2* seedlings carrying *G/NOS-GUS* (**I**) and *G-GATA/NOS101-GUS* (**J**) transgene. Error bars represents SD (n = 3). Error bars represents SD (n = 3). ** P≤0.01 for values significantly differ from WT in respective in respective tissues. All the above experiments were performed at least thrice with similar results.

Analysis of *G/NOS101* promoter suggests that *G/NOS101-GUS* was very weakly expressed in all the parts of the plant tested. Whereas leaf, stem and flower were more intensely stained in *atmyc2* **,** no difference in the promoter activity (if any) was detected between wild type and *atmyc2* in the roots (Figure 6A–D). Measurement of GUS activity also showed significant increase in *G/NOS101* activity in *atmyc2* mutants than wild type (Figure 6I) in all the organs tested except in roots. Analysis of *G-GATA/NOS101* promoter showed the activity of the promoter in leaf, stem, flower and root (Figure 6E–H) in wild type and *atmyc2* mutants. The quantification of the GUS activity measurements revealed that whereas the promoter activity was increased in stem in *atmyc2* mutants, it decreased in the flower and roots as compared to wild-type background (Figure 6J). No significant difference in the promoter activity was found in the leaf between wild-type and *atmyc2* mutants (Figure 6J).

## Discussion

The promoters vary depending upon the associated regulatory elements, specific sequence motifs and the choice of transcription start sites. LREs have been investigated in detail with context to their sequence, copy numbers, combinatorial effects, and also to some extent about their interacting protein partners. However, understanding the regulation of individual LRE by genetically and functionally defined light signaling components still remains largely unknown. In this study, we have shown the regulation of one of the least studied LREs, the Z-box, by two downstream signaling components SPA1 and MYC2, which predominantly work at two different wavelengths of light. We have demonstrated how high-irradiance light signals of different wavelengths can regulate the activity of Z-box containing promoters. We have observed that repression or induction of the activities of the Z-box containing promoters by light is regulated by proteins primarily responsive to their respective wavelengths of light.

Promoters are crucial for the controlled expression of genes in a spatio-temporal and stimulus specific manner. The traditional mutation and deletion analyses along with new high throughput technologies have enabled in identifying promoter and its regulatory elements, and thereby has helped investigating the mode of gene regulation. However, although quite a few number of cis-acting elements and the trans-acting factors involved in the light mediated transcriptional regulation have been reported, only few (G, GATA, GT1, Z-box) of them have been reported to play essential roles [28]–[30], [33], [35], [42]. Of late, the role of Z-box LRE in the regulation of transcription has been started to be unravelled in plants [38], [42], [43]. Transcription factors such as ZBF1/MYC2, ZBF2/GBF1 and ZBF3/CAM7 that specifically interact with the Z-box have been identified, and the functions of these transcription factors in light signaling pathways have also been demonstrated [14], [23], [25], [36], [37]. Interestingly, all these Z-box binding transcription factors have also found to be interacting with the G-box LRE. In this study, the Z- or G-box containing promoters are found to be regulated by ZBF1/MYC2 in somewhat similar fashion. Consistently, earlier studies have revealed that the Z- and G-box are functionally equivalent with context to ZBF2/GBF1 [36].

Interestingly, although SPA1 acts as a negative regulator of photomorphogenic growth, it is found to positively regulate the activity of *Z/NOS101* promoter mainly in the roots. Consistent with these results SPA1 has been reported to function positively for the lateral root development [23], [44]. However, SPA1 negatively regulates the activity of the Z-box containing *CAB1* minimal promoter in dark grown seedlings. Thus, the regulation of the Z-box by SPA1 shows contrasting effects with context to the promoter. It is worth mentioning here that although MYC2 is directly involved in the regulation of the Z- or G-box containing promoter, SPA1-mediated regulation is likely to be indirect. It has been shown earlier that the single element containing promoters may not mimic the regulation of the paired-element containing promoters or native promoters [28], [33], [42]. At least another molecule, SHW1, which functions as negative regulator for hypocotyl growth, but positive regulator for *CAB1* expression has been reported [45].

The Z- and G-box have been shown to be critical for the light-mediated induction of *CAB1* and *RBCS-1A* promoters, respectively [31], [32]. The Promoter-reporter analyses in this study demonstrate that MYC2 is a strong negative regulator of Z- and G-box containing promoters. The induction kinetics studies of *CAB1* native promoter further supports that MYC2 is a strong repressor of *CAB1* in both light and dark grown seedlings, and also during the transition from dark to light and vice versa. On the other hand, MYC2 plays both negative and positive regulatory roles in a tissue specific manner in the adult plants. MYC2 is a strong negative regulator of Z- and G-box containing promoters irrespective of promoter type (*Z/NOS101*, *CAB1-GUS*, *G/NOS101* and *G-GATA/NOS101*) in adult plants. However, MYC2 positively regulates *Z/NOS101* and *G-GATA/NOS101* promoter in the roots and flowers. MYC2 apparently does not regulate *CAB1* and *G/NOS101* promoters in the roots. It should be noted here that although soil grown plant roots are not exposed to light, the effect of light on the plant growth including root has well been documented [49], [50].

**Figure 7:**
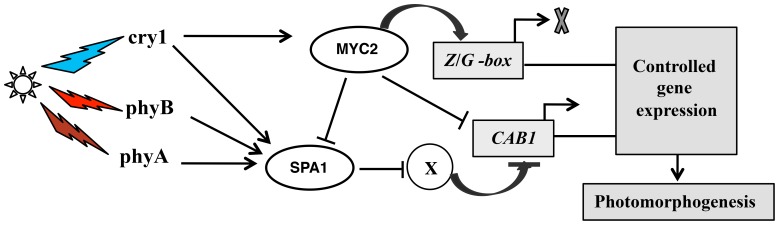
Mode of regulation of *Z*- and/or *G-*box containing promoters by MYC2 and SPA1. MYC2 inhibits the expression of *SPA1* [23]. MYC2 negatively regulates the Z- and/or G-box containing promoters irrespective of light quality by directly binding to the promoters. Whereas SPA1 positively regulates the Z-box containing promoter, it negatively regulates the activity of native *CAB1* in a wavelength independent manner through unknown regulatory protein (X) during photomorphogenesis.

The differential regulation of Z- and G-box containing promoters by MYC2 could be envisioned by multiple mode of actions. Firstly, differential stability and dynamics of the MYC2 protein in different tissue types. Second, the transcription factors (either positive or negative), which are directly or indirectly under the control of MYC2, may play crucial role in the differential regulation of these promoters in different tissue types. Third, Combinatorial interaction of bHLH and Myb transcription factors could be one plausible mode of regulation. Very recently, it has been shown that transcript and protein accumulation of MYC2 are regulated by circadian clock [46]. Also, TIME FOR COFFEE (TIC), a circadian clock component, acts as negative regulator of JA signaling pathway by degrading MYC2 protein [46]. Combinatorial interaction of bHLH and Myb transcription factors has been well documented for anthocyanin biosynthesis in maize [47], [48]. In conclusion, this study demonstrates that in modulation of photomorphogenesis, SPA1 and MYC2 can mediate the differential regulation of the Z- and G-box containing promoters (Figure 7) from early seedling to flowering plants.
